# Transcriptome‐wide association study identifies multiple genes and pathways associated with pancreatic cancer

**DOI:** 10.1002/cam4.1836

**Published:** 2018-10-18

**Authors:** Liuyun Gong, Dan Zhang, Yutiantian Lei, Yuanjie Qian, Xinyue Tan, Suxia Han

**Affiliations:** ^1^ Department of Oncology The First Affiliated Hospital College of Medicine, Xi'an Jiaotong University Xi’an Shaanxi China; ^2^ Department of Cell Biology and Genetics, School of Basic Medical Sciences Xi’an Jiaotong University Health Science Center Xi’an China

**Keywords:** pancreatic cancer, genome‐wide association study, transcriptome‐wide association study

## Abstract

**Aim:**

To identify novel candidate genes for pancreatic cancer.

**Methods:**

We performed a transcriptome‐wide association study (TWAS) analysis of pancreatic cancer (PC). GWAS summary data were driven from the published studies of PC, totally involving 558 542 SNPs in 1896 individuals with pancreatic cancer and 1939 healthy controls. FUSION software was applied to the PC GWAS summary data for tissue‐related TWAS analysis, including whole blood, peripheral blood, adipose, and pancreas. The functional relevance of identified genes with PC was further validated by Oncomine, STRING, and CluePedia tool.

**Results:**

Transcriptome‐wide association study analysis identified 19 genes significantly associated with PC, such as LRP5L (*P* value = 5.21 × 10^‐5^), SOX4 (*P* value = 3.2 × 10^‐4^), and EGLN3 (*P* value = 6.2 × 10^‐3^). KEGG pathway enrichment analysis detected several PC‐associated pathways, such as One carbon pool by folate (*P* value = 1.60 × 10^‐16^), Cell cycle (*P* value = 1.27 × 10^‐7^), TGF‐beta signaling pathway (*P* value = 4.64 × 10^‐6^). Further comparing the 19 genes with previously identified overexpressed genes in PC patients found one overlapped gene SOX4.

**Conclusion:**

We identified some novel candidate genes and pathways associated with PC. Our results provide novel clues for the genetic mechanism studies of pancreatic cancer.

## 1 INTRODUCTION

Pancreatic cancer(PC) is a deadly disease, which leads to an estimated 227 000 deaths per year worldwide.^1^ The term pancreatic cancer contains both exocrine and endocrine tumors of pancreas. More than 90% of PCs originate from ductal epithelium.^2^ Risk factors for this malignant disease include smoking, advanced age, family history of chronic pancreatitis, male sex, diabetes, obesity, non‐O blood group, occupational exposures, diets high in meat and low in vegetables, and probable Helicobacter pylori infection and periodontosis.^1^

Although the cause of PC is multifactorial, the most important risk factors are smoking and family history.Nearly 20% of pancreatic tumors are caused by smoking, and cancers from smokers suffer more genetic mutations than those from nonsmokers.^1^ Genetic factors play important roles in the development of pancreatic cancer. Multiple genetic studies have been implemented and identified some susceptibility genes for PC, such as KRAS, BRCA1, BRCA2, PALB2, FANCC, FANCG, ATM, CDKN2A, PRSS1, SPINK1, TERT, NR5A2, ZNRF3, and SMC2.^3^ However, the genetic risk of PC explained by the reported candidate loci was limited, indicating the existence of undiscovered susceptibility loci for PCs.

Genome‐wide association studies (GWAS) are an efficient tool for genetic mechanism studies of complex diseases. Expression quantitative trait loci (eQTLs) are a group of important regulatory loci, which can regulate gene expression levels.^4^ In recent years, integrative analysis of GWAS data and eQTLs annotation information is rapidly becoming a standard approach for explore the genetic basis of disease susceptibility.^5^ Nowadays, transcriptome‐wide association study (TWAS) analysis was proposed to utilize extensive published GWAS data. TWAS analysis adopts pre‐computed gene expression weights together with disease GWAS summary statistics to estimate the association of each gene to diseases.^6^, ^7^ TWAS showed a high efficiency for identifying novel causal genes of complex diseases.^8^, ^9^, ^10^, ^11^

In this study, we conducted a tissue‐related TWAS for PC, considering whole blood, peripheral blood, adipose, and pancreas. TWAS was first applied to a large‐scale GWAS data to detect novel susceptibility genes associated with PC. The functional relevance of identified genes with PC was further validated by Oncomine, STRING, and CluePedia tool.

## 2 METHODS

### 2.1 GWAS summary datasets of PC

A large‐scale GWAS summary data of pancreatic cancer were used in this study.^7^ Briefly, this GWAS comprised of 1896 individuals with pancreatic cancer and 1939 controls from 12 prospective cohorts and a hospital‐based case‐control study. Samples were excluded based on low completion rates (<98%) and unexpected inter‐ or intra‐study duplicates. Commercial platform Illumina Hap500 Infinium genotyping assay was used for genome‐wide SNP genotyping. GLU, Genotyping Library and Utilities, was used for data analysis and management. The association between single SNPs and pancreatic cancer was tested by Logistic regression. SNPs were excluded based on low call rates (<90%). Detailed information of cohorts, genotyping, and quality control approaches can be found in the published studies.^7^

### 2.2 Gene expression datasets

The over‐expression genes in PC patients were driven from the Oncomine database.^12^ Oncomine (https://www.oncomine.org) is a cancer microarray database and web‐based data‐mining platform for facilitating discovery from genome‐wide expression analyses. Differential gene expression was identified by comparing major types of cancer to respective normal tissues.^12^, ^13^

### 2.3 TWAS of pancreatic cancer

FUSION software was applied to the PC GWAS summary data for tissue‐related TWAS analysis, including whole blood, peripheral blood, adipose, and pancreas. TWAS analysis uses pre‐computed gene expression weights together with disease GWAS summary statistics to calculate the association of every gene to disease.^14^ The genetic values of expression were computed one probe set at a time using SNP genotyping data located 500 kb on either sides of the gene boundary. For this study, the gene expression weights of whole blood, peripheral blood, adipose, and pancreas were driven from the FUSION website (https://gusevlab.org/projects/fusion/).^14^ The genes with significant and suggestive association signals were identified at *P* value <3.73 × 10^‐6^ after strict Bonferroni correcting and *P* value <0.05, respectively.

### 2.4 PPI network and pathway enrichment analysis

The functional relevance of identified genes with PC was further validated byOncomine, STRING, and CluePedia tool. STRING (Search Tool for the Retrieval of Interacting Genes) (https://string-db.org/cgi/input.pl) is an online tool designed to evaluate the protein‐protein interaction (PPI) network.^15^, ^16^ The CluePedia, a plugin of Cytoscape software, is a tool for searching potential genes associated with the certain signaling pathway by calculating linear and nonlinear statistical dependencies from experimental data.^17^, ^18^ The PPI (protein‐protein interaction) network of significant genes identified by TWAS was constructed by STRING. We also analyzed the signaling pathways of these significant genes by STRING, then verified, and visualized them by CluePedia. The candidate pathways were identified at *P* value <0.05.

## 3 RESULTS

### 3.1 TWAS analysis results

A total of 13 412 genes were analyzed by TWAS in this study. After strict Bonferroni correcting, TWAS identified 19 genes with suggestive association signals (*P* value <0.05) (Table 1), such as LRP5L (*P* value = 5.21 × 10^‐5^), SOX4 (*P* value = 3.2 × 10^‐4^), EGLN3 (*P* value = 6.2 × 10^‐3^), TXNDC15 (*P* value = 6.5 × 10^‐3^), PPP2R2A (*P* value = 9.7 × 10^‐3^), and E2F3（*P* value = 4.4 × 10^‐2^).

**Table 1:** List of genes identified by TWAS for PC (*P* value<0.05)

Gene	Chromosome	GWAS SNP ID	EQTL ID	TWAS *P* value	Comparative tissue
KCNK5	6	rs2162840	rs228815	4.67E‐02	Adipose
RP11‐65J3.1	9	rs10819503	rs17457362	4.67E‐02	Adipose
PPP2R2A	8	rs6557943	rs10495	9.70E‐03	Adipose
PPFIBP2	11	rs1552462	rs10839816	3.47E‐02	Adipose
GEMIN4	17	rs4985584	rs3744743	1.71E‐02	Adipose
NIPA2	15	rs17524529	rs7168115	3.82E‐02	Peripheral blood
RNASEH2B	13	rs7985384	rs9568490	3.98E‐02	Peripheral blood
FARS2	6	rs6920599	rs2224391	3.11E‐02	Whole blood
E2F3	6	rs4712465	rs874447	4.36E‐02	Whole blood
SOX4	6	rs12530233	rs12530233	3.22E‐04	Whole blood
MTHFD1L	6	rs13201018	rs13201018	1.09E‐02	Whole blood
F2R	5	rs4438855	rs153311	3.95E‐02	Whole blood
TXNDC15	5	rs6889478	rs12656013	6.46E‐03	Whole blood
NDUFA3	19	rs254260	rs254262	1.50E‐02	Whole blood
CRISPLD2	16	rs1566465	rs8057431	3.21E‐02	Whole blood
CHSY1	15	rs4965799	rs2012387	1.78E‐02	Whole blood
EGLN3	14	rs1680699	rs1769603	6.24E‐03	Whole blood
IQSEC3	12	rs12582189	rs2291926	1.92E‐02	Whole blood
LRP5L	22	rs5761251	rs13057190	5.21E‐05	Whole blood

### 3.2 PPI network and pathway enrichment analysis

**Figure 1:**
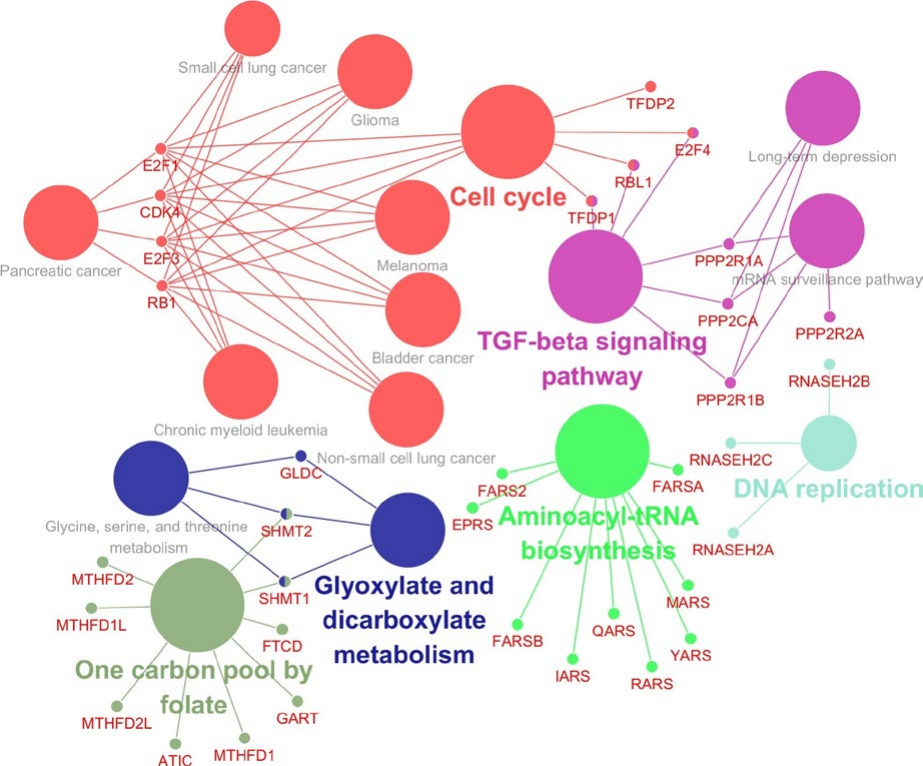
Visualization of KEGG pathways in of the 19 PC‐associated genes identified by TWAS. Functionally grouped network with terms as nodes linked based on their kappa score level (≥ 0.3). Only the label of the most significant term per group is shown. The node size represents the enrichment significance of the term. Functionally related groups partially overlap. The color gradient shows the gene proportion of each cluster associated with the term

The PPI network and signaling pathways of the 19 PC‐associated genes were generated by STRING (Table 2). The top 10 candidate pathways identified by the STRING tool were one carbon pool by folate (*P* value = 1.60 × 10^‐16^), aminoacyl‐tRNA biosynthesis (*P* value = 4.64 × 10^‐13^), metabolic pathways (*P* value = 2.07 × 10^‐7^), cell cycle (*P* value = 1.27 × 10^‐7^), TGF‐beta signaling pathway (*P* value = 4.64 × 10^‐6^), bladder cancer (*P* value = 1.71 × 10^‐5^), purine metabolism(*P* value = 2.75 × 10^‐5^)，non‐small cell lung cancer (*P* value = 5.95 × 10^‐5^)，pancreatic cancer (*P* value = 7.72 × 10^‐5^)，glioma(*P* value = 7.72 × 10^‐5^). The results showed that relevant genes of PC were CDK4, E2F1, E2F3, and RB1 (Table 2). Then, we verified and visualized the linkage of genes with PC by CluePedia (Figure 1). The genes enriched to PC were coincident with the results from STRING.

**Table 2:** List of top 15 KEGG pathways in 19 association genes of PC (*P* value<0.05)

Pathway description	Count	STRING P value	Gene ID	CluePedia *P* value
One carbon pool by folate	9	1.60E‐16	*ATIC,FTCD,GART,MTHFD1,MTHFD1L,MTHFD2,* *MTHFD2L,SHMT1,SHMT2*	19.0E‐16
aminoacyl‐tRNA biosynthesis	9	4.64E‐13	*EPRS,FARS2,FARSA,FARSB,IARS,MARS,QARS,* *RARS,YARS*	34.0E‐11
Metabolic pathways	18	1.27E‐07	*ADSL,ATIC,CHSY1,EPRS,FTCD,GART,GLDC,* *MTHFD1,MTHFD1L,MTHFD2,MTHFD2L,* *NDUFA3,PAICS,PFAS,PPAT,QARS,SHMT1,SHMT2*	0.0026
Cell cycle	8	1.27E‐07	*CDK4,E2F1,E2F3,E2F4,RB1,RBL1,TFDP1,TFDP2*	18.0E‐7
TGF‐beta signaling pathway	6	4.64E‐06	*E2F4,PPP2CA,PPP2R1A,PPP2R1B,RBL1,TFDP1*	0.00004
Bladder cancer	4	0.000171	*CDK4,E2F1,E2F3,RB1*	0.00052
Purine metabolism	6	0.000275	*ADSL,ATIC,GART,PAICS,PFAS,PPAT*	0.0032
Non‐small cell lung cancer	4	0.000594	*CDK4,E2F1,E2F3,RB1*	0.0014
Pancreatic cancer	4	0.000772	*CDK4,E2F1,E2F3,RB1*	0.0024
Glioma	4	0.000772	*CDK4,E2F1,E2F3,RB1*	0.0024
Tight junction	5	0.000783	*CDK4,PPP2CA,PPP2R1A,PPP2R1B,PPP2R2A*	0.0035
Melanoma	4	0.000968	*CDK4,E2F1,E2F3,RB1*	0.0024
Chronic myeloid leukemia	4	0.000968	*CDK4,E2F1,E2F3,RB1*	0.0022
Small cell lung cancer	4	0.00194	*CDK4,E2F1,E2F3,RB1*	0.0034
mRNA surveillance pathway	4	0.00199	*PPP2CA,PPP2R1A,PPP2R1B,PPP2R2A*	0.0023

### 3.3 Identification of overexpressed genes in SCLC

We identified overexpressed and down‐expressed genes in PC by comparing the PC samples to normal tissues in Oncomine online database. The top 1% genes overexpressed and down‐expressed in 78 PC samples were shown in Appendix S1. After comparing the genes identified by TWAS with the overexpressed and down‐expressed genes detected by Oncomine, we found one overlapped gene SOX4, which was overexpressed in PC patients. Furthermore, the significant SNP rs12530233 of SOX4 gene was also an eQTL, suggesting the functional importance of rs12530233 in the development of PC.

## 4 DISCUSSION

Pancreatic cancer is the main cause of cancer death worldwide. The cause of pancreatic cancer is complex and multifactorial. Smoking, advanced age, and family history of chronic pancreatitis are main risk factor for PC.^1^ Most patients with pancreatic cancer remain asymptomatic until the tumor metastasizes to other tissues and organs.^19^ As there is no standard program for screening patients at early‐stage of PC, it is necessary to find more effective susceptibility gene for PC prevention.

Consistent with the result of TWAS, previous studies have reported 4 of the 19 genes (PPP2R2A, E2F3, KCNK5, and SOX4) play important roles in the development of PC,^20^, ^21^, ^22^, ^23^ and 2 of the 19 genes (CHSY1 and EGLN3) have been proved associated with PC via bioinformatics methods.^25^, ^26^ SOX4 is significantly associated with PC and overexpressed in PC patients. Furthermore, it is interesting that the significant SNP rs12530233 of SOX4 gene is an eQTL, suggesting the importance of rs12530233 in the dysfunction of SOX4 expression regulation during the development of PC. Previous research has verified that SOX4 is expressed in the early processes of PC tumorigenesis and suggested that SOX4 might function as a master transcription factor in PC formation.^24^ Further studies, such as fine mapping and RNA sequencing of SOX4, are needed to confirm our finding and clarify the potential mechanism of SOX4 involved in the development of PC.

Another susceptibility gene E2F3, is a transcription factor family, plays an important role in cellular proliferation, apoptosis, and differentiation.^27^ MiR‐210 is induced by hypoxia and expressed in the development of PC. Chen et al^28^ have suggested that E2F3 may be potential miR‐210 targets in PC. A systems‐level analysis of the scale‐free GMCs network taken by Rajamani et al^29^ identified that E2F3 is associated with PC progression.

It has been known that PPP2R2A encodes an alpha isoform from the regulatory subunit B55 subfamily (B55α) and can selectively control Akt phosphorylation.^30^ Intriguingly, Hein et al^21^ have reported that PPP2R2A promotes PC development by maintaining hyperactivity of multiple oncogenic signaling pathways, including ERK, Wnt as well as AKT. In addition, Shen et al^22^ have found PPP2R2A was significantly higher expressed in SH‐PAN cells than DT‐PCa cells and proved that decreased expression of PPP2R2A inhibited the development and progression of PC.

KCNK5 (also known as TASK‐2 or K2P5.1) has been shown to be the volume sensitive K (+) channel in cells.^31^ KCNK5 is expressed in the kidney, liver, stomach, small intestine, colon, and pancreatic acinus. An electrophysiological study indicated that KCNK5 was expressed in human pancreatic ductal adenocarcinoma cell line, and the pH‐sensitive K2P subunits coded by KCNK5 were shown to be expressed in pancreatic.^20^

Transcriptome‐wide association study analysis identified several novel candidate genes for PC, such as RP11‐65J3.1, PPFIBP2, GEMIN4, NIPA2,RNASEH2B, FARS2, MTHFD1L, F2R, TXNDC15, NDUFA3, CRISPLD2, IQSEC3, and LRP5L. As is known to all, few efforts have been paid to investigate the potential roles of them in the formation of PC. Further biological experimental studies are required to confirm our finding and clarify the potential roles of novel candidate genes in the development of PC.

Pathway enrichment analysis detected several candidate pathways for PC, some of which has been reported to be implicated in the development of PC directly or indirectly. For instance, it has been demonstrated that TGF‐beta signaling pathway in pancreatic cancer can be utilized in targeted therapy clinical trials.^32^ Another interesting pathway is one carbon pool by folate, which is associated with influenced PC incidence.^33^ Researchers have also proved that aminoacyl‐tRNA biosynthesis, metabolic pathways, cell cycle, tight junction, purine metabolism, and mRNA surveillance pathway are existed in PC cells and related to occurrence and development of PC.^33^, ^34^, ^35^, ^36^, ^37^, ^38^ The rest of these signaling pathways are solid tumor directly. Like bladder cancer, lung cancer, glioma, melanoma, chronic myeloid leukemia as well as PC.

In summary, we conducted a tissue‐related TWAS analysis and identified some novel candidate genes and pathways associated with PC. Our results provide novel clues for clarifying the genetic mechanism of PC. Further biological studies are warranted to confirm our findings and reveal the potential mechanism of identified genes and pathways involved in the development of PC.

## CONFLICT OF INTEREST

The authors declare no conflict of interests.
